# Identifying CpG methylation signature as a promising biomarker for recurrence and immunotherapy in non–small-cell lung carcinoma

**DOI:** 10.18632/aging.103517

**Published:** 2020-07-28

**Authors:** Ruihan Luo, Jing Song, Xiao Xiao, Zhengbo Xie, Zhiyuan Zhao, Wanfeng Zhang, Shiqi Miao, Yongyao Tang, Longke Ran

**Affiliations:** 1 Department of Bioinformatics, The Basic Medical School of Chongqing Medical University, Chongqing, China; 2 Department of Endocrine and Breast Surgery, The First Affiliated Hospital of Chongqing Medical University, Chongqing, China; 3 Information Center Department, Chongqing Medical University, Chongqing, China; 4 Molecular and Tumor Research Center, Chongqing Medical University, Chongqing, China

**Keywords:** CpG methylation signature, recurrence, non–small-cell lung carcinoma, tumor mutation burden, immunotherapy

## Abstract

Epigenetic alterations are crucial to oncogenesis and regulation of gene expression in non–small-cell lung carcinoma (NSCLC). DNA methylation (DNAm) biomarkers may provide molecular-level prediction of relapse risk in cancer. Identification of optimal treatment is warranted for improving clinical management of NSCLC patients. Using machine learning algorithm we identified 4 recurrence predictive CpG methylation markers (cg00253681/ART4, cg00111503/KCNK9, cg02715629/FAM83A, cg03282991/C6orf10) and constructed a risk score model that potently predicted recurrence-free survival and prognosis for patients with NSCLC (P = 0.0002). Integrating genomic, transcriptomic, proteomic and clinical data, the DNAm-based risk score was observed to significantly associate with clinical stage, cell proliferation markers, somatic alterations, tumor mutation burden (TMB) as well as DNA damage response (DDR) genes, and potentially predict the efficacy of immunotherapy. In general, our identified DNAm signature shows a significant correlation to TMB and DDR pathways, and serves as an effective biomarker for predicting NSCLC recurrence and response to immunotherapy. These findings demonstrate the utility of 4-DNAm-marker panel in the prognosis, treatment decision-making and evaluation of therapeutic responses for NSCLC.

## INTRODUCTION

Lung cancer is a prominent global health issue and economic burden, with an estimated 40.9 million disability-adjusted life-years in 2017 [1]. Non-small cell lung carcinoma (NSCLC) is the most frequent tumor type of lung cancer, accounting for approximately 87% of lung cancer cases, most of which were diagnosed with advanced stage [2]. In recent years, NSCLC still poses a huge health threat to all mankind in the case of high morbidity and mortality as well as poor prognosis due to late disease diagnosis and not being eligible for curative surgery. Even though traditional adjuvant platinum-based chemotherapy or target therapies have been beneficial for advanced resected tumors, most still have a high relapse risk [3–5]. One potential explanation for these clinical phenomena could be that traditional prognosis indicators are not helpful in making optimal therapy strategies for NSCLC patients with different states of recurrence risk. It would be of great significance to investigate a better prognostic molecular signature to predict recurrence and determine the patients with NSCLC who might benefit most from adjuvant therapies.

It has been well-known that DNA methylation (DNAm), as an epigenetic modulator, regulates gene expression in cancer [6]. The pattern of DNAm alterations which are locus dependent, includes hypo- and hyper-methylation of oncogene and tumor suppressor genes respectively, and has been proved to correlate with oncogenesis, progression and treatment [7, 8]. Methylome profiling carries several benefits: lots of altered CpG sites within DNA methylation target region, relatively stable methylation aberrance, and higher clinical sensitivity in cancer detection [9]. Besides, epigenetic therapy (low doses of DNMTi) exerts durable anti-tumor effect, avoiding acute cytotoxicity [10]. Prior studies showed well-renowned SEPT9 in colorectal cancer (CRC) [11] and MGMT in CRC with metastasis [12] were sensitive and effective methylation markers for diagnosis and prognosis. DNAm locus aberrance was also proved in lung tumor tissues and the epigenetic alterations might associate with prognosis of patients with stage I lung cancer [13]. Nevertheless, little is known about molecular function of specific DNAm markers or a methylation panel, and few of which are with clinical utility and widely accepted for NSCLC patients. Therefore, investigation into clinically effective and reliable DNAm signature is warranted for evaluating relapse risk of NSCLC.

Immune checkpoint blockades (ICBs) therapies in advanced NSCLC patients demonstrated prominent durable response, and higher tumor mutation burden (TMB) correlated to improved relapse-free survival, durable objective response as well as elevated clinical efficacy [14]. Both mutation load and methylation loss accumulate during mitotic cell division [15], and chromosome instability may arise from mutations in a DNA methyltransferase gene [16]. Hence, it’s imperative that the DNAm signature and its contribution to immunotherapy responding patient stratification in NSCLC be explored to discover routine and potent biomarkers for identification of potential responders to ICBs treatment.

In this study, we initially identified 4 CpG biomarkers associated with recurrence of NSCLC. Base on TCGA NSCLC cohort comprised of lung adenocarcinomas (LUAD) and lung squamous cell carcinomas (LUSC), a promising DNAm-based risk score model predictive of relapse was constructed and then validated in the other 3 datasets. We further explored molecular mechanism and clinical utility of the DNAm signature. At last we investigated the relevance of the combined DNAm panel with TMB and clinical response to ICBs.

## RESULTS

### Patient and clinical characteristics in NSCLC cohorts

**Figure 1:**
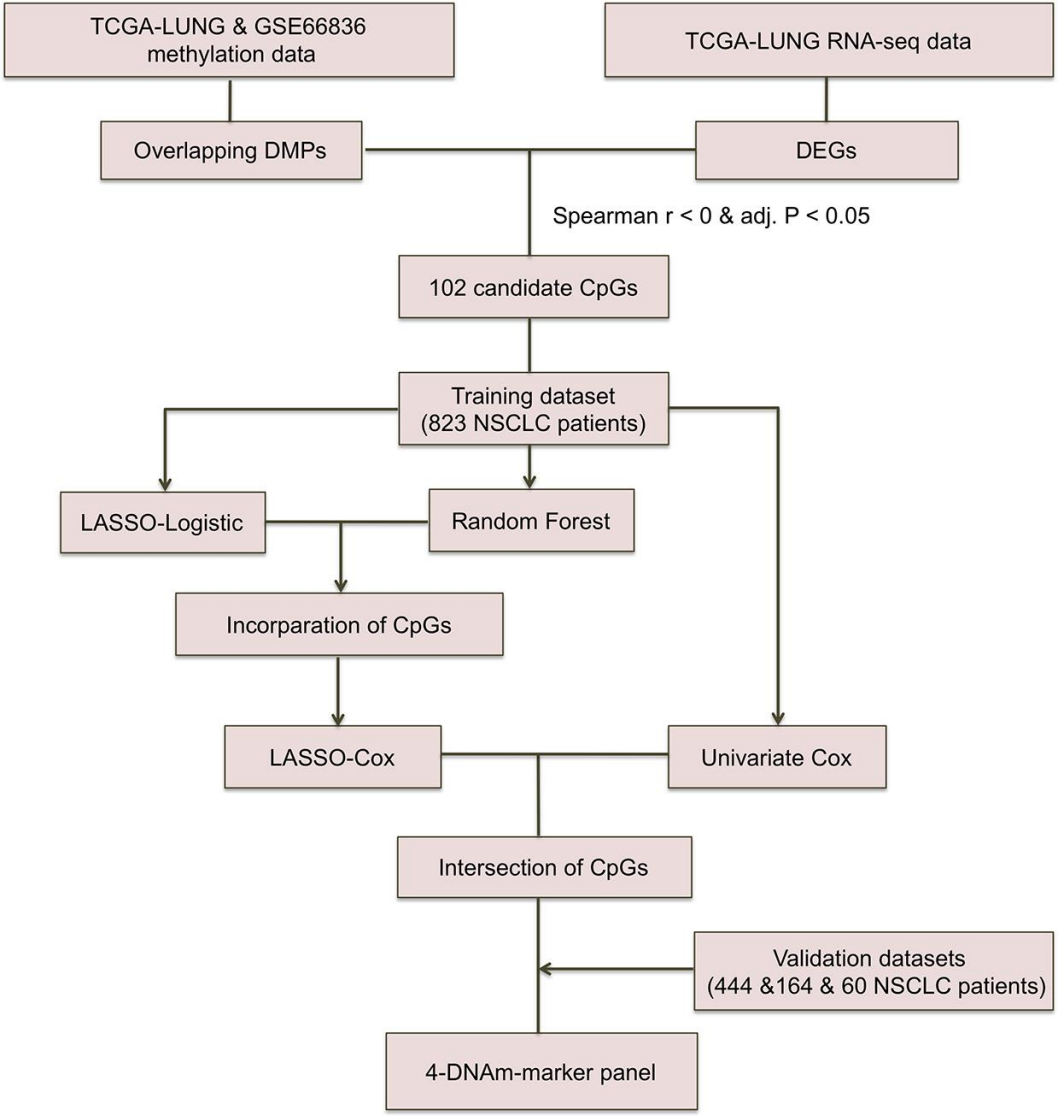
**Workflow chart of CpG marker selection. Two DNA methylation (DNAm) datasets and TCGA RNA-seq dataset were used for identifying 102 candidate CpG markers.** Based on recurrence-free survival data of the training cohort (823 TCGA NSCLC patients), LASSO-Logistic and Random Forest methods were applied to identify recurrence associated CpG markers. With the incorporation of CpGs identified by two methods above, LASSO-Cox were implemented to select robust DNAm signatures. Using the CpGs overlapped in results of univariate Cox and LASSO-Cox models, the 4-DNAm-marker panel was finally identified and verified in validation cohorts. adj.P: Bonferroni corrected P.

NSCLC patients included were mainly derived from TCGA LUNG Cancer cohort, GSE39279, GSE66836 and GSE119144 cohorts with clinical characteristics presented in Supplementary Table 1. DNAm data for a total of 827 TCGA NSCLC and 60 GSE119144 tumor samples were available at initial analysis, whereas RFS information of only 662 TCGA NSCLC patients (393 non-recurrence and 271 recurrence tumor tissue samples) and 59 GSE119144 NSCLC patients (10 non-recurrence and 49 recurrence tumors) was complete and available for analysis in training and validation phase, respectively. Beyond all that, we also utilized DNAm data of GSE66836 (164 LUAD samples) from GEO repository. Work flowchart of DNAm prognostic marker selection was depicted in Figure 1.

### DNA methylation and gene expression profiles in NSCLC

**Figure 2:**
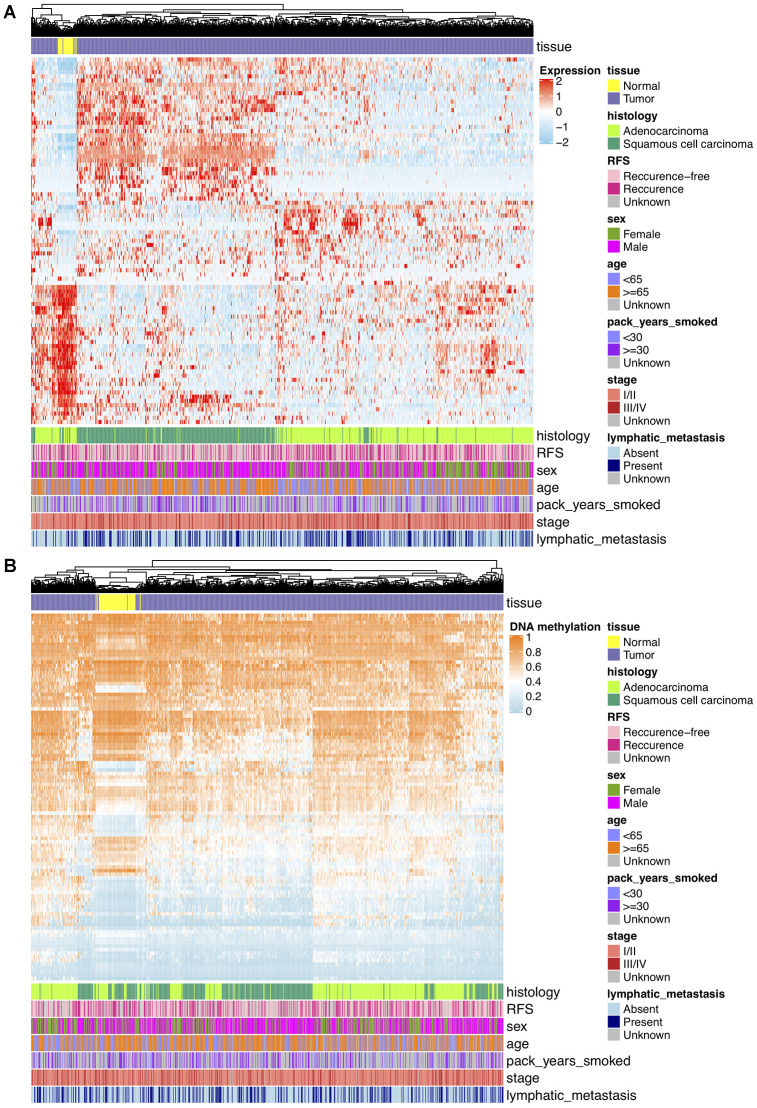
(**A**) Hierarchical clustering of 87 unique DEGs that potentially regulated by changes in DNAm levels at 102 selected loci based on TCGA NSCLC gene expression data (821 tumor and 28 normal samples). Clinical and demographic features, including age, sex, pack-years smoked, histology, stage, lymphatic metastasis and clinical outcome (RFS, recurrence-free survival status). High expression, red; low expression, skyblue. (**B**) Hierarchical clustering of 102 significantly DMPs between NSCLC (n=827) and normal (n=74) samples. Hopomethylated CpGs, skyblue; hypermethylated CpGs, orange.

Analyses of DNAm differences between NCSLC tumors and normal lungs were conducted on TCGA and GSE66836 datasets, revealing that DNA methylation in 11641 overlapping CpGs representing 5359 unique genes were of significant aberration. To keep consistent with loci in GSE39279 and GSE119144 datasets, DMPs were further filtered and 9367 consistent CpGs among training and validation sets were retained. For transcriptomic profiling, differential expression analysis on TCGA RNA-seq data that matched with DNAm profiles showed 1717 significant DEGs, including 1282 upregulated and 435 downregulated genes, and from which 270 potentially hyper- and hypo-methylated genes nearby DMPs mentioned above were identified and used for further shrinking CpGs. After Spearman’s correlation test (r < 0, Bonferroni corrected P < 0.05) on association between DNAm and mRNA expression levels, 102 CpGs representing 87 unique DEGs finally yielded. These DMPs were deemed as biologically meaningful where DNAm changes probably epigenetically regulated and negatively correlated to reference gene expression nearby. On the basis of results above, unsupervised hierarchical clustering of 87 DEGs separated 849 TCGA NSCLC samples into tumor and normal subgroups, revealing 53 genes were upregulated and 34 genes were downregulated in tumor tissues (Figure 2A). Unsupervised clustering analysis of 102 significant DMPs also presented a clear distinction between tumor and normal samples of TCGA DNAm data, in which 57 DMPs were hypomethylated while 45 were hypermethylated in NSCLC tissues (Figure 2B).

### Identification of recurrence predictive CpGs for NSCLC

**Figure 3:**
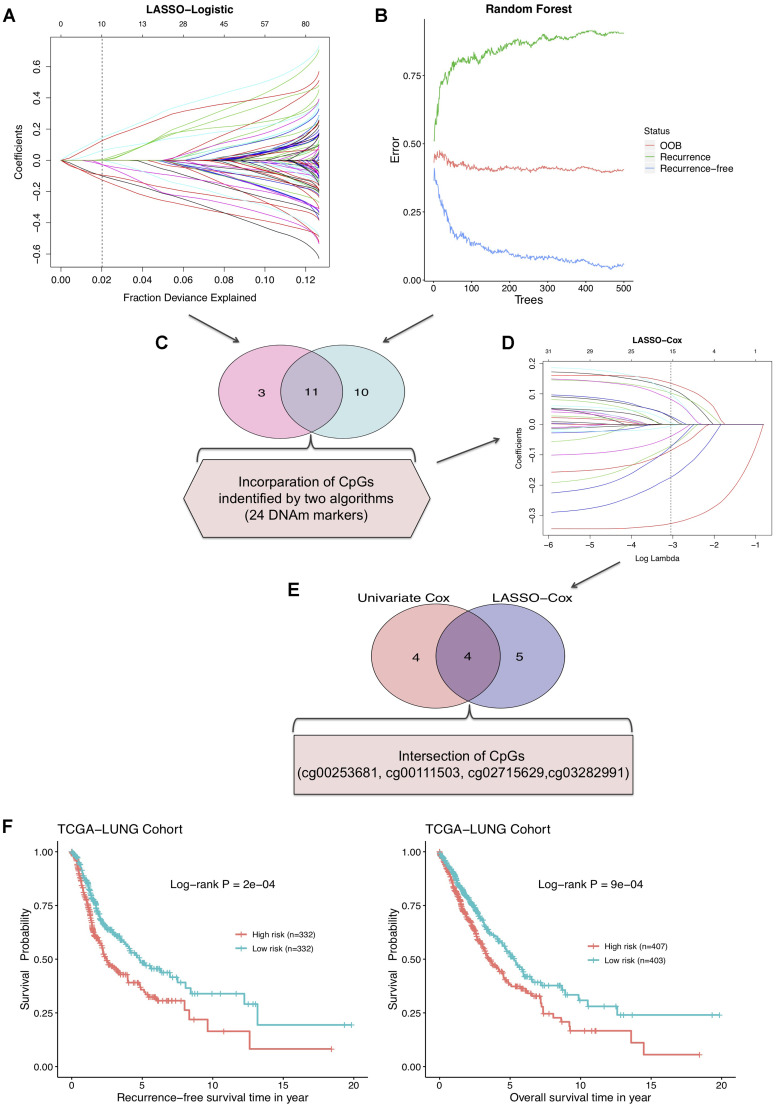
(**A**) LASSO-Logistic and (**B**) Random Forest methods applied to identify recurrence associated DNAm markers in training cohort. (**C**) A total of 11 overlapping CpGs and 24 combined CpGs in two algorithms. (**D**) LASSO-Cox analysis performed to select robust relapse predictive CpGs. (**E**) Four final identified CpGs in the intersection of univariate Cox and LASSO-Cox results. (**F**) The RFS curve (left) and overall survival curve (right) of training cohort based on 4-DNAm-maker panel.

To screen out the DNAm markers predictive of relapse risk for NSCLC patients, methylation values of DMPs in 664 TCGA tumor samples were included into following analyses with machine learning algorithms. We firstly implemented two methods: LASSO-Logistic regression and Random Forest on modeling 102 aforementioned DMPs for narrowing down markers, identifying 14 and 21 CpGs respectively (Figure 3A, 3B, Supplementary Figure 1, Supplementary Table 2). A total of 11 CpGs were overlapped in results of two algorithms, and 24 CpGs unioned together were then incorporated into LASSO-Cox model, yielding 9 robust prognostic CpG markers (Figure 3C, 3D, Supplementary Table 2). Plus, univariable Cox regression analysis was performed with relapse-free survival data of training set, and 8 CpGs were screened out, with 4 most significant CpG markers identified simultaneously by LASSO-Cox and univariate Cox methods (Figure 3E, Supplementary Table 2, Supplementary Figure 1). By combining CpGs selected from LASSO-Cox and univariate Cox models, 13 predictive biomarkers were obtained. Subsequently, multivariable analysis was conducted on clinicopathological factors in combination with the 13 CpGs (Supplementary Table 3). Using DNAm profile multiplied by coefficients of the multivariate Cox regression model, based on 4 ultimate CpGs (Table 1), a risk score model generated for prediction of recurrence in NSCLC. The median of risk score was set as the cutoff value and NSCLC patients were divided into high-risk group (risk score ≥ -0.0416) and low-risk group (risk score < -0.0416) (Supplementary Figure 2).

**Table 1:** Characteristics of four prognostic CpG markers and their coefficients on the basis of multivariate Cox proportional hazard model.

**Marker**	**Ref Gene**	**Coefficient**	**Hazard Ratio**
cg00253681	ART4	0.339	1.4
cg00111503	KCNK9	-0.337	0.71
cg02715629	FAM83A	-0.138	0.87
cg03282991	C6orf10	0.037	1.04

We further investigated the potential of risk score model in NSCLC prognostic prediction. Survival analysis on the combined risk score showed that RFS probabilities of NSCLC patients in high- and low-risk groups were of salient difference, and the 4-DNAm-marker panel also presented favorable potential in predicting overall survival (OS) in TCGA NSCLC cohort (Figure 3F). Moreover, according to results of multivariable survival analysis, the risk score was an independent prognostic indicator for NSCLC patients and significantly associated with both RFS and OS (Supplementary Figure 3, Supplementary Table 4). AJCC stage system was extensively used for clinical prognostic evaluation, whereas this DNAm-based risk score was found to show better discriminative power of relapse status than clinical stage and other four separate CpGs in training and validation sets (Supplementary Figure 4).

### Clinical and molecular features, and mutations associated with the prognostic DNA methylation signature

**Figure 4:**
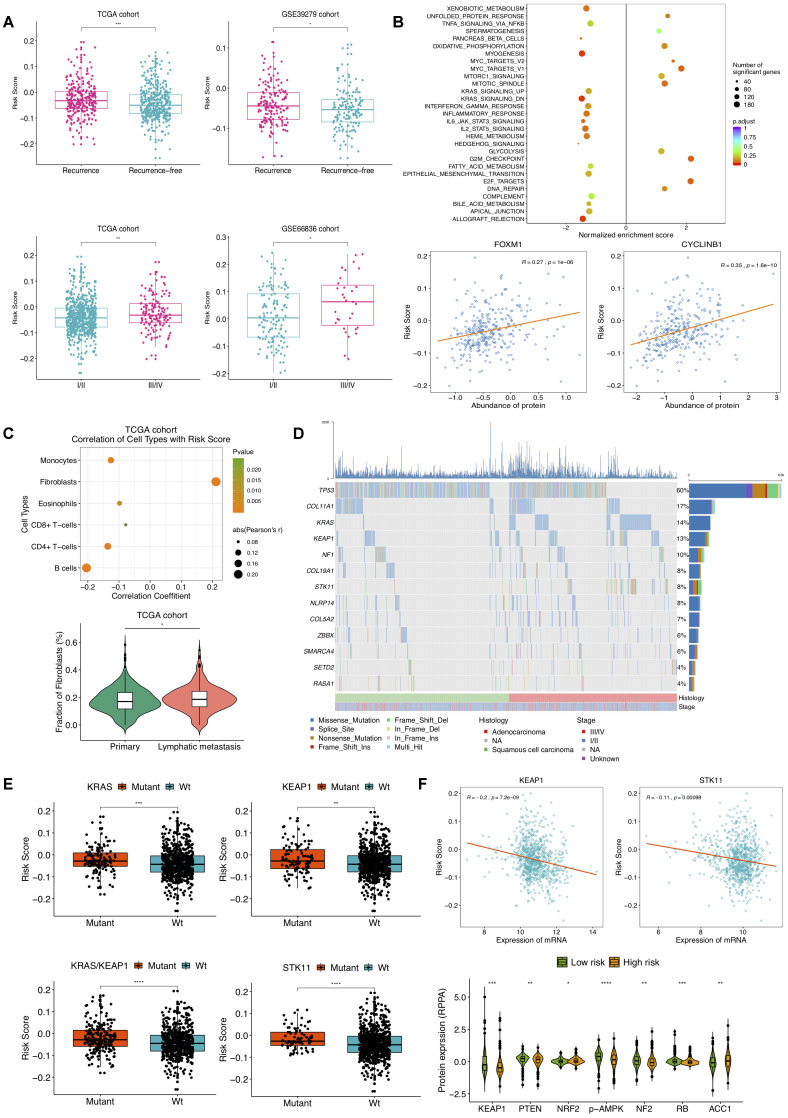
**Selected clinical, molecular features and mutations associated with DNAm-based risk score.** (**A**) Relationship of clinical characteristics and the DNAm signature. DNAm-based risk score stratified by different stages and recurrence status from TCGA NSCLC patients (left) and from GSE39279 (top right) and GSE66836 cohorts (bottom right). (**B**) GSEA on a set of hallmark gene signatures revealing the impact of the identified DNAm signature on cell cycle, proliferation and immune-related pathways (top); DNAm-based risk score strongly correlated to expression of FOXM1 and CYCLINB1 protein (bottom). (**C**) Relevance of estimated cell-type fractions with risk score (top); The abundance of fibroblasts in NSCLC patients relates to lymphatic metastasis status (bottom). (**D**) Mutation profile of TCGA NSCLC samples showing 13 SMGs of which mutational proportion correlated with DNAm signature. (**E**) Association of the DNAm signature with mutation in genes. DNAm-based risk score stratified by mutations in KRAS, KEAP1, STK11 and KRAS/KEAP1 co-mutations. (**F**) Correlation of DNAm signature with representative gene (top) and protein (bottom) expression.

We next assessed the correlation of the DNAm signature with clinical characteristics and molecular features. The association between the risk score and tumor progression was found to be positive in TCGA NSCLC patients. Risk scores of tumors with recurrence were significantly higher than those without, and TCGA NSCLC samples at different stages had significant different relapse risk (Figure 4A). Besides, the similar results were also obtained on GSE39279 and GSE66836 datasets (Figure 4A). Then, we sought to investigate the molecular implications hidden behind current correlations. GSEA on two TCGA NSCLC sample subgroups divided by predictive recurrence risk revealed that high-risk group endowed significant enrichment of gene signatures mainly related to E2F targets, G2M checkpoint and MYC targets V1 (Figure 4B). According to RPPA analysis of LUAD tumors based upon TCGA repository, higher risk score was significantly correlated with higher expression of FOXM1 and CYCLINB1 proteins. FOXM1, a transcription factor upstream of CYCLINB1 (a G2/M transition marker), has been reported to associate with cell proliferation, whose overexpression is related to metastasis and poor prognosis in ovarian [17] and clear cell renal carcinoma [18], which suggested high cellular cycling and cell proliferation in NSCLC patients with high recurrence risk score. Also, epigenetic treatment of combining DNA-demethylating agents with histone deacetylase inhibitors decreased MYC signaling, exerting robust anti-tumor effect in NSCLC [19]. Notably, prominent correlations with risk score were also observed for pathways including allograft rejection, inflammatory response and IL2-STAT5 signaling (Figure 4B). Tumor microenvironment (TME) is deemed as intricate and dynamic in exacerbating and inhibiting tumor cell proliferation, migration, invasion and metastasis. On account that salient results of pathway enrichment analysis on the DNAm signature primarily consisted in cell cycle, proliferation and immune-related pathways, we were then committed to evaluating DNAm-based risk score in the context of TME.

Within TME, component system activation plays an important role in the connection of inflammation and anti-tumor immune response as well as oncogenesis [20]. Prior studies have proposed that epigenetic changes can regulate inflammation and immune signaling [19]. We thus tried to assess cellular composing in NSCLC based on DNAm profiles of TCGA and GSE66836 cohorts. After implementing HEpiDISH function, we found escalating risk score was associated with an increased fibroblasts and a reduced immune-cell fraction. In Figure 4C presented significant correlations between the DNAm signature and estimated compositions of B-cells, CD4+ T-cells, CD8+ T-cells, eosinophils, monocytes, and fibroblasts in TCGA NSCLC samples. There were no significant correlations for recurrence risk score with assessed enrichments of neutrophils and NK cells (data not shown). We also noted that the fraction of fibroblasts was associated with lymphatic metastasis (Figure 4C), suggesting fibroblasts in TME might induce tumor inflammation and boost NSCLC progression as well as metastasis [21]. Likewise, strong associations of B-cells, CD4+ T-cells, monocytes, eosinophils and fibroblasts abundance with DNAm-based risk score were observed in GSE66836 cohort (Supplementary Figure 5). These findings indicated that the higher fibroblasts fraction and lower infiltrating levels of immune cells might emerge in NSCLC patients with high recurrence risk, which were also consistent with previous reports demonstrating significant association between low density of CD8 +T-cells and poor RFS in papillary thyroid cancer [22].

Subsequently, we linked the DNAm signature to mutations in genes. Based on mutation profiles of TCGA NSCLC tumors, several SMGs identified by MutsigCV v.1.41 and also correlated with DNAm-based risk score were presented in Figure 4D. A notable correlation of our risk score with somatic mutations in gene KRAS, KEAP1, STK11, and co-occurring KRAS/KEP1A mutations was found in NSCLC (Figure 4E). Furthermore, we found a significant association of this DNAm signature with mRNA expression of KEP1A and STK11 (Figure 4F). Higher NRF2, ACC1 and lower KEAP1, PTEN, p-AMPK, NF2 and RB protein abundance were observed in high-risk group (Figure 4F). Loss-of-function type mutation in KEAP1 results in activation of NRF2, which accelerates lung cancer cell growth [23]. Combined loss of PTEN and KEAP1 promotes LUAD formation in mice model [24]. In current study, high DNAm-based risk score was connected to low expression of STK11, low AMPK activation as well as STK11 mutation, indicating the mTOR activation of TCGA NSCLC samples in high-risk group [25]. Elevated ACC1 levels in patients with hepatocellular carcinoma were correlated to vascular invasion and disease recurrence [26]. The inactivation of NF2 in malignant Pleural Mesothelioma with mTOR activity aberrantly upregulated, fails to inhibit cell proliferation, leading to a poor prognosis [27]. Absence of RB in a mouse model of LUAD was demonstrated to drive disease progression and metastasis [28].

### Methylation signature correlates to TMB and DDR genes

**Figure 5:**
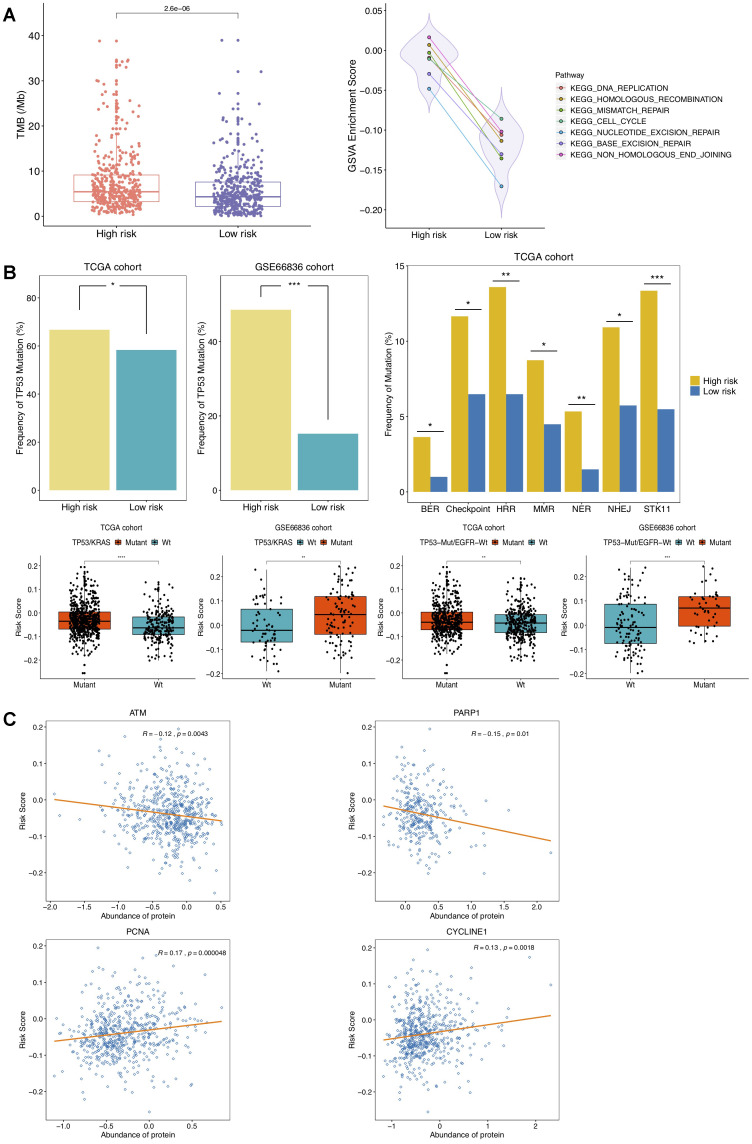
**Correlation of DNAm-based risk score with TMB, cell cycle, DNA damage response (DDR) genes.** (**A**) Left, TMB estimation of TCGA NSCLC patients in high- and low-risk group; right, GSVA presenting DDR pathways significantly enriched in high risk group (adjusted P < 0.01). (**B**) Estimated frequencies of mutations in TP53 (top left), STK11 and DDR genes (top right), TP53/KRAS co-mutations and TP53-Mut/EGFR-Wt mutations (bottom) under different recurrence risk status. (**C**) Protein expression of ATM, PARP1, PCNA and CYCLINE1 associated with DNAm-based risk score in TCGA cohort.

In recent time, tumor mutation burden (TMB) has been well-reported as an emerging and promising indicator for clinical benefit of immunotherapy [29]. DNA hypomethylation was proposed to induce chromosomal aberrations, prompting chromosome instability [30]. Based upon nonsynonymous coding mutations in somatic mutation data matched with methylome data of TCGA NSCLC tumors, we then quested for the connection between DNAm signature and TMB. It turned out to be noteworthy higher TMB in high-risk group (Figure 5A), suggesting DNAm-based risk score might predict immune evasion of NSCLC to some extent. Methylation loss was linked to an increase in mutation density and cell cycle gene expression by mitotic cell division [31]. Methylation status of four selected DMPs and expression levels of nearby reference genes at four CpG sites in our risk score model were also correlated with TMB (Supplementary Figure 6). We further inspected the underlying biological structure with respect to our DNAm signature to inquire into interpretation for distinct TMB between high- and low-risk groups. GSVA were performed on recurrence risk status, which revealed that gene sets of cell cycle, DNA replication, homologous recombination, nonhomologous end-joining, mismatch repair, base excision repair and nucleotide excision repair were significantly upregulated in high-risk group (Figure 5A). All these observations implied relevance of cell proliferative processes activation for high risk status, and also implicated that this DNAm signature might have connection to alterations in cell cycle, DNA replication and DDR pathway genes. Increased TMB and improved efficacy of ICBs were proposed to independently associate with alterations in DDR genes [32]. In order to determine whether DNAm signature might underlie and account for differential TMB by influencing molecular mechanisms analyzed above, we subsequently aimed at figuring out the correlation of DDR-related genes and DNAm-based risk score.

The related gene signatures involved in six of aforementioned DDR pathways were listed in Table 2 and defined as BER, NER, HRR, MMR, NHEJ and Checkpoint genes [33], respectively. Also, six kinds of co-mutations in DDR genes above were analyzed in this study. It has been well-established that TP53 mutation accelerates cell cycle and DNA replication, and TP53/KRAS co-mutation exhibits a remarkable increased mutational loads and owns a potential of predicting response to immunotherapy in LUAD [34]. In this study, higher frequency of TP53 mutation in high-risk group was observed in patients from TCGA NSCLC as well as GSE66836 cohort (Figure 5B), indicating NSCLC tumors with higher risk score were more susceptible to yielding DNA replication errors. Additionally, the presence of TP53 alterations without EGFR or STK11 mutations is revealed to identify LUAD patients who respond to anti-PD1 therapies [35]. Significant associations of risk scores with TP53/KRAS co-mutation, combination of TP53-Mut/EGFR-Wt mutation as well as somatic mutations in STK11 and in other co-analyzed DDR genes were also identified in this study (Figure 5B). The mTORC1-S6K pathway is aberrantly activated due to STK11 loss, leading to DNA damage response defects and prompting genome instability [36]. The mutated ATM is reported to correlate with genomic instability and ATM predominantly responds to DNA double-strand breaks (DSB) [37, 38]. What’s more, we found significant associations of the DNAm signature with waning protein abundance of ATM, PARP1, and raised expression of PCNA and CYCLINE1 protein (Figure 5C). Overexpressed CCNE1, an oncogene encoding cell cycle protein CYCLINE, induced DNA replication stress by premature S phase entry, leading to genomic instability [39]. All analyses above suggested that changes of DNAm patterns in NSCLC might predispose epigenetically impacting on TMB by mediating alteration in cell-cycle regulating and DDR genes, resulting in more neoantigens formation and changes of the tumor antigenicity.

**Table 2:** Gene list for six related DNA damage repair response pathways.

**Pathway**	**Genes**
BER	NEIL3, PARP1, PCNA
Checkpoint	ATM, TIMELESS, TP53
HRR	BRCA1, BRCA2, XRCC2
MMR	MLH1, MSH3, MSH4
NER	ERCC1, ERCC6, TCEB3
NHEJ	DCLRE1C, PRKDC

*BER, base excision repair; HRR, homologous recombination repair; MMR, mismatch repair; NER, nucleotide excision repair; NHEJ, non-homologous end-joining.

### NSCLC patients with high risk score present favorable clinical benefit to immunotherapy

**Figure 6:**
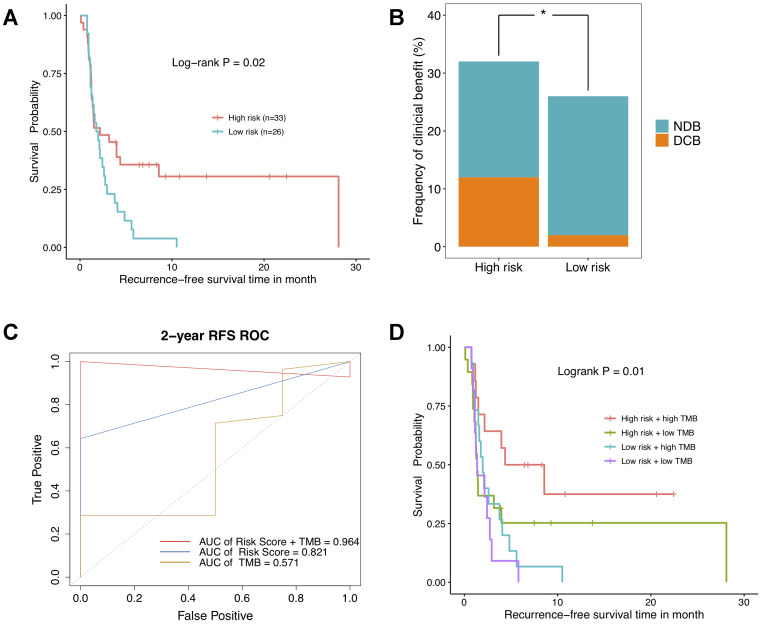
**The relationship between DNA methylation signature and clinical response to immunotherapy investigated in GSE119144 cohort.** (**A**) Relapse-free survival curves comparing high-risk with low-risk groups in NSCLC patients received anti-PD-1/PD-L1 therapies, according to DNAm-based risk score from validation set. (**B**) Proportion of clinical benefit to immunotherapy in the indicated groups stratified by our DNAm signature (DCB: durable clinical benefit and NDB: no durable benefit). (**C**) Time-dependent ROC curves for DNAm-based risk score, TMB, and risk score combined with TMB. (**D**) RFS curves of NSCLC patients with combinations of risk score and TMB.

To go further, the relevance of DNAm-based risk score with response to ICBs was investigated on GSE119144 cohort. We observed RFS of immunotherapeutic patients in high-risk group was significantly superior to that of those in low-risk group (Figure 6A). Besides, a greater percentage of NSCLC patients with high risk score owned a durable clinical benefit, while most of low-risk group patients had no durable clinical benefit (Figure 6B). Notably, the combination of DNAm signature and TMB saliently improved the ability to predict clinical responses to immunotherapy (AUC = 0.965, Figure 6C). As was revealed in Kaplan–Meier curves, NSCLC patients separated by this combination of two variables harbored significantly different clinical outcomes (P = 0.01, Figure 6D). These results implicated high risk score and high mutational burden might represent robust biomarkers to determine best responders to ICBs treatments. And anyway, the clinical application effect deserves further research and being corroborated in larger cohorts with longer follow-up data.

## DISCUSSION

In present study, based on methylation and transcriptome data as well as corresponding clinical information of training and validation cohort, we initially selected 4 DMPs predictive of NSCLC relapse by applying machine learning methods, and built a risk score model comprised of 4 CpG markers. To demonstrate clinical utility of the 4-DNAm-marker panel, training and validation NSCLC cohorts could be effectively divided into high-risk and low-risk groups of tumor relapse. In this manner, it’s convenient and beneficial for clinicians to conduct individualized medical treatment and heath management.

Given that higher DNAm-based risk score linked to adverse clinical outcomes, we assumed our DNAm signature might underlie and facilitate development and progression in NSCLC. To better understand implications of DNAm signatures in clinical events of NSCLC, we then sought to explore the biological function, molecular mechanism as well as hidden compounding somatic variants, and also evaluated cell-type composition from an epigenetic perspective. In this work, we observed the recurrence predictive risk score of TCGA NSCLC patients to be correlated with clinical characteristics, fractions of immune cell infiltrates, molecular features in the layers of both mRNA and protein expression, as well as somatic mutations in genes involved in specific signaling pathways. Interestingly, a remarkable association between the DNAm signature and TMB was identified in TCGA NSCLC cohort. Additionally, we noted DNAm-based risk score was connected to several DDR genes, which could be a favorable interpretation of high TMB in high-risk group. Ultimately, this DNAm signature was demonstrated to be a potential biomarker that predicted clinical response to immunotherapy and survival of NSCLC.

This is, to the best of our knowledge, a comprehensive investigation on NSCLC by integrating TCGA NSCLC data for DNAm, mutations, clinical features, expression of mRNAs and proteins, which provides insight into molecular mechanism, prognostic, and therapeutic implications. We propose that the 4-DNAm-marker panel acts as an effective biomarker for predicting metastasis and recurrence in NSCLC. The hematogenic metastasis formation can be characterized by the extravasation of leukocytes and tumor cells [40]. In the context of TME, it’s of necessity to investigate individual cell-type enrichments for reflecting tumor-immune interactions. Prior studies have established the notion that CD4+ /CD8+ T-cells are capable of recognizing cancer antigens and positively associated with favorable RFS in ovarian cancer [41]. We noted that higher risk score was correlated to increased fibroblasts and reduced leukocyte fractions, indicating 4-DNAm-marker panel might have the potential to serve as an indicator of characterizing immune infiltration landscape in NSCLC. The combination epigenetic therapy (Aza + ITF-2357) induced the increased levels of CCL5, a secreted chemokine attracting functional lymphocytes, restraining tumor growth with increased number of CD8+ T cells in mice model of NSCLC [19]. Our observations also suggested that several immune cell subtypes in NSCLC were indispensable for tumor progression.

Previous studies put forward that genetic characterization revealed by WES or targeted sequencing might have influence over therapeutic options, assessment of treatment response and patients’ prognosis in some solid cancers [42]. Investigations on targeted therapies directed towards several somatically altered pathways are thus essential for medical decision and clinical implementation. We presented that mutational patterns of TCGA NSCLC tumors were associated with the DNAm signature, in which a strong correlation of higher DNAm-based risk score with recurrently mutated driver genes KRAS, KEAP1 and STK11 was observed in this study. We also noted that high risk score was correlated to co-mutations in KRAS/KEAP1, and associated with expression of KEAP1, STK11 mRNA as well as several other key protein abundance. KEAP1 deletion contributes to tumor aggressiveness, metastasis, and increases radioresistance in LUSC [43]. Somatic oncogenic point mutations in KRAS were proposed to be crucial to progression and drug resistance in 90% of patients with pancreatic ductal adenocarcinoma, a highly metastatic disease with a high mortality rate [44]. A higher incidence of metastasis emerged in KRAS-mutated CRC patients, whose relapse pattern depends on the KRAS mutational status with down-regulation of p-MAPK signaling prompting and forming distant lung metastasis [45]. Somatic KEAP1 mutation leads to activation of the NRF2 pathway, and NSCLC patients with KEAP1 mutation in addition to an activating KRAS mutation were demonstrated to have a shorter duration of platinum-based chemotherapy and a worse prognosis than other patients with KRAS-mutant [46]. We speculated that epigenetic patterns might describe genetic alterations in NSCLC tumors and further reflect tumor aggressiveness and resistance to therapy. Our identified DNAm signature of TCGA NSCLC tumors were also observed to connect with recurrence-associated and especially cell-cycle related gene signatures that induce tumor metastasis for many tumor types, suggesting that epigenetic changes may impact on activation of cell cycle and disease progression.

The unmethylated status of FOXP1 indicates durable response to ICB therapies and improved survival of a subset of NSCLC patients, and could correlate to validated and up-to-date biomarkers such as mutational load [29]. Earlier studies proposed that DNA replication stress leads to genomic instability in cancer, which can be characterized by the rates of high mutation as well as epigenetic perturbation, especially for DNA methylation loss [15, 47]. TMB of TCGA NSCLC patients was found prominently associated with the DNAm-based risk score in our study. We also revealed underlying mechanisms hidden behind this correlation. It turned out that DNAm signature also interplayed with genomic alterations in DDR pathways, implicating NSCLC patients in high-risk group potentially endowed endogenous replication stress and genomic instability. In the light of results analyzed above, we speculate that DNAm-based risk score may contribute to the identification of those individuals who will be more susceptible to immunotherapy and predicting clinical efficacy of ICBs. More recently, several studies have proposed DNAm alterations might work as biomarkers of immune evasion with higher predictive power than mutation burden [48], but the more specific DNAm signature remained to be elucidated. The sensitive measure of TMB estimation demands WES or a minimum gene panel size of 150 [49]. Clinically, our 4-DNAm-maker panel, by contrast, can avoid the high cost of WES or deep sequencing and be cost-efficient in practice. Combining epigenetic treatment, depletion of Myc reverses immune invasion of lung cancer, enhancing effectiveness of immune checkpoint treatment [19]. It’s conceivable that a promising epigenetic therapy, possibly coupled with immunotherapies will generate remarkable clinical benefit.

Several limitations to our study should be noted: Firstly, another independent NSCLC cohort which is performed with DNAm assay and also received ICBs therapy with detailed follow-up data will be required to validate our observation. Second, this study was based on DNAm data of NSCLC tissue biopsy and it’s preferable that our results could be also validated by ctDNA methylation analysis which is relatively noninvasive and clinically feasible. Thirdly, more detailed biologic mechanisms of final selected markers remains to be investigated on laboratory experiments.

To sum up, the combined DNAm signature (cg00253681, cg00111503, cg02715629, and cg03282991) is a reliable biomarker for predicting clinical benefit to ICBs treatments and recurrence in NSCLC. Our study shed light on the implications of epigenetic modulation in disease recurrence prediction, treatment strategy selection and evaluation of responses to immunotherapy.

## MATERIALS AND METHODS

### NSCLC patient data

The Cancer Genome Atlas (TCGA) LUNG Cancer, GSE66836, GSE39279 and GSE119144 cohorts included in this study were derived from online public data repository, with NSCLC patients who received neoadjuvant chemotherapy excluded. For DNAm data, a total of 901 TCGA NSCLC samples were available using the Illumina Infinium HumanMethylation450 platform, including 827 tumor tissues and 74 non-tumor tissues. DNAm level 3 data were obtained at the website: https://tcga.xenahubs.net. In addition to TCGA data, we also analyzed GSE66836 dataset (164 LUADs, 19 normal lungs), GSE39279 and GSE119144 cohorts that recruited 444 and 60 NSCLC patients respectively. Three methylation microarray datasets and corresponding clinical data were downloaded from Gene Expression Omnibus (GEO) database. DNAm levels of 4 datasets above were all measured by beta values for each CpG probe, which ranged from 0 (completely unmethylated) to 1 (completely methylated). TCGA LUNG Cancer gene expression data consisted of 1116 samples, including 1007 NSCLC tissues and 109 normal tissues. RNA sequencing (RNA-seq) level 3 expression data (normalized read counts) and related clinical data were available at https://tcga.xenahubs.net. TCGA NSCLC somatic mutation data comprised of somatic variant calls in TCGA-LUAD (n=562) and TCGA-LUSC (n=486) cohorts were retrieved from https://gdc.xenahubs.net.

### Analysis of epigenetic profiles

Right at the beginning, we aimed at identifying significant differentially methylated positions (DMPs) in TCGA NSCLC tumor versus normal lung tissues. TCGA DNAm level 3 data for 485578 CpGs were parsed into the limma package [50] with limma function implemented to assess the differential DNAm. A robust DMP was defined as containing CpG that yielded a Benjamini-Hochberg (BH) adjusted P < 0.05, without “NA” for the average beta in each group. To obtain more reliable DMPs, the same analysis steps were conducted on Methylation 450K Beadchip data (normalized beta values) of GSE66836, then overlapping DMPs of 2 datasets were retained for subsequent analyses. The DMPs with log_2_ fold change < 0 were regarded as hypomethylated and > 0 were regarded as hypermethylated. To pursuit meaningful epigenetic profiling, 450k data in our study were annotated by R package lluminaHumanMethylation450kanno.ilmn12.hg19.

### Gene expression data and reverse phase protein array profiling

Subsequently, analysis of aberrant gene expression in 821 TCGA NSCLC tissues compared with 28 matched normal lung tissues (consistent with samples in DNAm data) was performed by edgeR package. A threshold value of false discovery rate (FDR) < 0.05 and |log_2_ fold change| > 1 was used for screening significant differentially expressed genes (DEGs). The upregulated genes (FDR < 0.05, log_2_ fold change >1) and downregulated genes (FDR < 0.05, log_2_ fold change < -1) were further utilized for screening hypomethylated CpGs showing higher expression and hypermethylated CpGs showing lower expression, respectively. To integrate mRNA expression and epigenetic profile, the associations between DEGs and differentially methylated loci located within 2 kb of transcript start site were then assessed by Spearman correlation. The Bonferroni corrected P < 0.05 and r < 0 were used as cut-off criteria for further filtration.

As is described in previous researches, it’s preferable that we performed analysis of mRNA expression incorporated with protein expression profiles for reflecting biological complexity more systematically and comprehensively [51]. To assess protein levels of TCGA cohort, we downloaded the reverse phase protein array (RPPA) profiles of 687 NSCLC samples (including 362 LUAD and 325 LUSC samples) from MD Anderson (http://app1.bioinformatics.mdanderson.org/tcpa/_design/basic/index.html).

### Clinical data and predictive modeling

To identify potential recurrence predictive methylation markers for NSCLC patients, four aforementioned cohorts were included for prognostic prediction using candidate CpGs in combination with corresponding clinical characteristics (adjuvant radiation and chemotherapy, histology, sex, age, stage, pack-years smoked, lymphatic and distant metastasis).

In training phase, TCGA LUNG Cancer cohort was used as the training dataset, in which 664 NSCLC specimens have detailed recurrence-free survival (RFS) information. First, Random Forest and Least Absolute Shrinkage and Selection Operator (LASSO) Logistic models were applied to select recurrence-related DNAm markers. Meanwhile, univariate cox regression was performed to identify CpG markers associated with RFS of NSCLC patients. According to theoretical basis of LASSO method, Cox proportional hazards regression model with LASSO penalty generates regression coefficients that strictly equal to 0, removing some of variables with lower weights on the purpose of data dimension reduction, and preventing overfitting resulting from collinearity of the covariates [52]. To further identify more significant markers, LASSO-Cox method was subsequently implemented on the incorporation of CpGs selected by LASSO-Logistic and Random Forest algorithms. Next, the consistent CpGs identified by univariate Cox and LASSO-Cox methods were retained and then incorporated into a multivariable Cox regression model. Eventually, GSE39279, GSE66836 and GSE119144 were taken as validation datasets, in which GSE119144 cohort was consisted of 60 NSCLC patients who underwent anti-PD-1/PD-L1 ICBs treatments, with complete follow-up information available about 59 samples.

### Gene set enrichment analysis and gene set variation analysis

To investigate the association between NSCLC recurrence risk status predicted by the DNAm-based risk score and gene signatures, using R package “clusterprofiler” [53] we implemented gene set enrichment analysis (GSEA) on TCGA mRNA expression data. Two NSCLC subgroups (High risk vs. Low risk) were divided according to median risk score, on which fold change values were calculated and used for GSEA on a set of 50 hallmark signatures [54]. Additionally, the “GSVA” package [55] was utilized for identifying pathways most related to the DNAm signature. The gene set variation analysis (GSVA) was performed with a set of 186 KEGG pathway signatures. Gene signatures with adjusted P < 0.05 were considered significant differentially enriched. Two reference gene sets above were downloaded from the Molecular Signature Database (MSigDB): http://software.broadinstitute.org/gsea/msigdb/index.jsp.

### Somatic mutation profiling and cell-type fraction estimating

Highly confident somatic variants, including single nucleotide variation and short insertion/deletion polymorphism, from a total of 1048 TCGA NSCLC samples were integrated in this study. Using MutsigCV v.1.41 [56] somatic variant calls from TCGA NSCLC tumors were analyzed for significantly mutated genes (SMGs); among them, SMGs with FDR value below 0.05 were retained. The visualized part and summarization for MAF files of TCGA NSCLC whole-exome sequencing (WES) data were implemented by R package maftools [57]. To evaluate the tumor mutation burden (TMB), we computed the number of non-synonymous somatic mutations in coding region for each tumor sample. Based on a primary reference containing fibroblasts and a secondary reference that contains 7 immune cells subtypes (B-cells, NK cells, CD4+ and CD8+ T-cells, monocytes, neutrophils and eosinophils) [58], we applied the HEpiDISH method on DNAm profiles of TCGA and GSE66836 datasets by R package “EpiDISH” to infer individual cell-type fractions for NSCLC patients included. The correlations between the DNAm signature and estimated enrichments of cell types were subsequently investigated.

### Statistical analyses

All statistical analyses were implemented using R version 3.6.2. Unsupervised hierarchical clustering was conducted by package ComplexHeatmap in R using the DNA methylation levels of selected DMPs as well as the mRNA expression levels of DEGs nearby those CpGs. The R package “randomForest” and “glmnet” were used for Random Forest and LASSO model. The 10-fold cross validation was performed on two algorithms to optimize Random Forest model with minimum misclassification rate and obtain the optimal lambda values (the minimum lambda value) in LASSO models. Kaplan-Meier curves analyses and log-rank tests were performed by the survminer package. Furthermore, the survival package was used for survival analysis with DNAm signature and clinicopathological parameters combined in a multivariable Cox proportional hazards regression model. To estimate the performance of CpG markers in training and validation sets, we conducted receiver operating characteristic (ROC) curve analyses using pROC package. In addition, time depended ROC analysis was performed by the survival ROC package. We performed the Wilcoxon test followed by multiple testing using the BH correction approach to figure out difference of DNAm-based risk score between mutant subgroups, between related clinical-factor groups, and difference of TMB estimation in high-risk vs. low-risk group. Spearman correlation analysis was used to assess the relationship of DNAm signature with estimated cell-type fractions, DNA damage response (DDR) genes and proteins. The mutation frequencies in DDR genes between high- and low-risk groups were compared using Chi-square (χ2) test. For all statistical tests, two-tailed P < 0.05 denoted statistical significance, which is indicated by *, P < 0.05, **, P < 0.01, ***, P < 0.001, ****, P < 0.0001.

### Data accessibility

The methylation chip data for NSCLC samples included in our study are accessible through GEO accession number GSE66836, GSE119144 and GSE39279. RPPA data are available on MD Anderson TCGA database. All TCGA NSCLC data can be accessed at https://tcga.xenahubs.net.

## Supplementary Material

Supplementary Figures

Supplementary Tables
