# Supplementary material for: Identifying CpG methylation signature as a promising biomarker for recurrence and immunotherapy in non–small-cell lung carcinoma
Source: Aging (Albany NY). 2020 Jul 28;12(14):14649–76. doi: 10.18632/aging.103517 (PMC7425482; doi:10.18632/aging.103517)
Supplement: Supplementary Figures [file aging-12-103517-s002..pdf]

SUPPLEMENTARY FIGURES

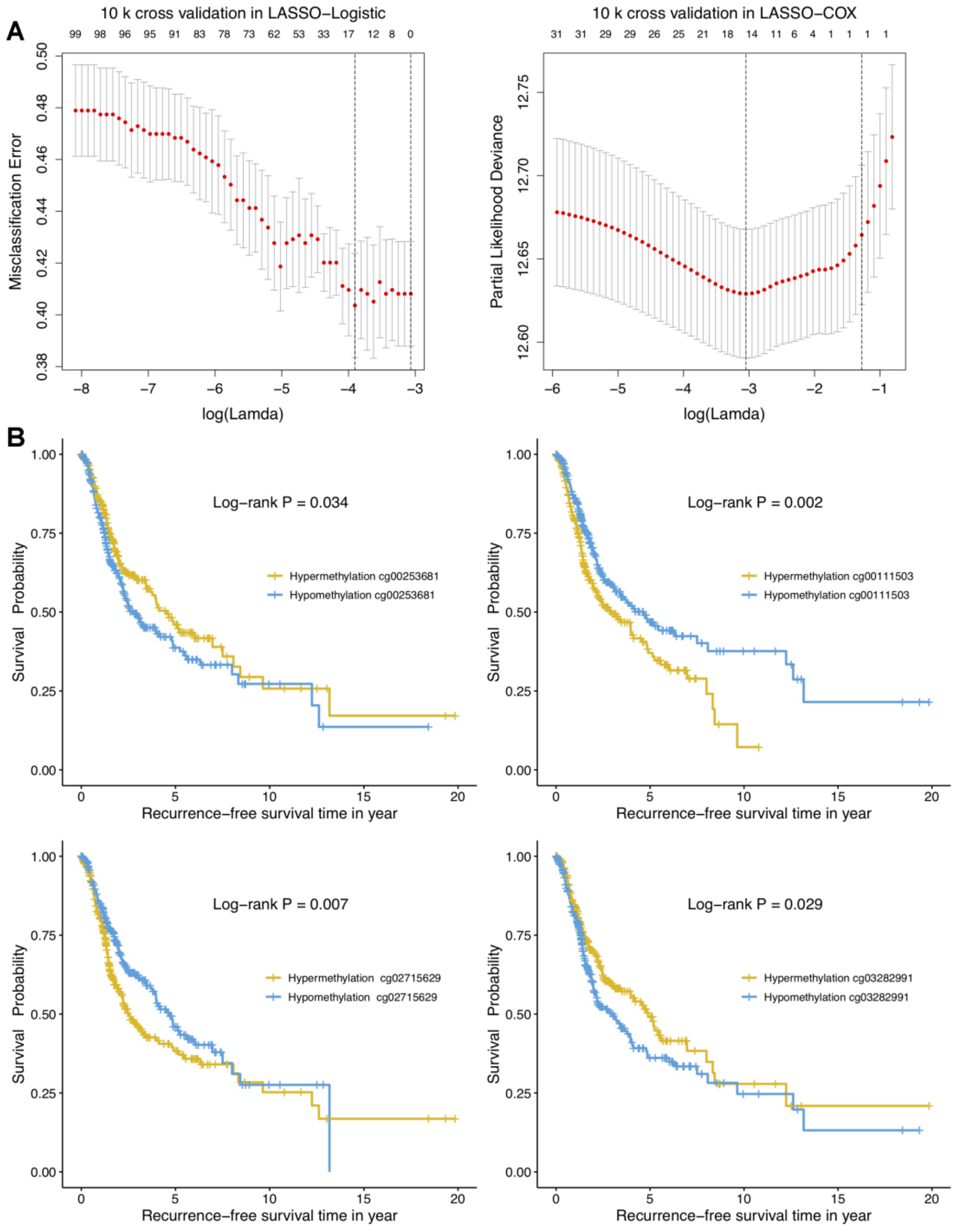

Supplementary Figure 1. (**A**) Ten-fold cross validations performed for obtaining optimal parameter lambda (λ) in LASSO-Logistic (left) and LASSO-Cox analysis (right). The dotted vertical lines were plotted at values of log (λ) by minimum criteria and 1-Standard Error criteria in two LASSO models, respectively. The optimal values of λ were determined by minimum criteria where two dotted vertical lines were drawn in Figure 3A and Figure3D, and thus 14 and 9 CpG markers with nonzero coefficients were screened out. (**B**) Kaplan-Meier curves of four final selected CpGs present their correlation with recurrence and prognostic prediction of NSCLC patients in training cohort.

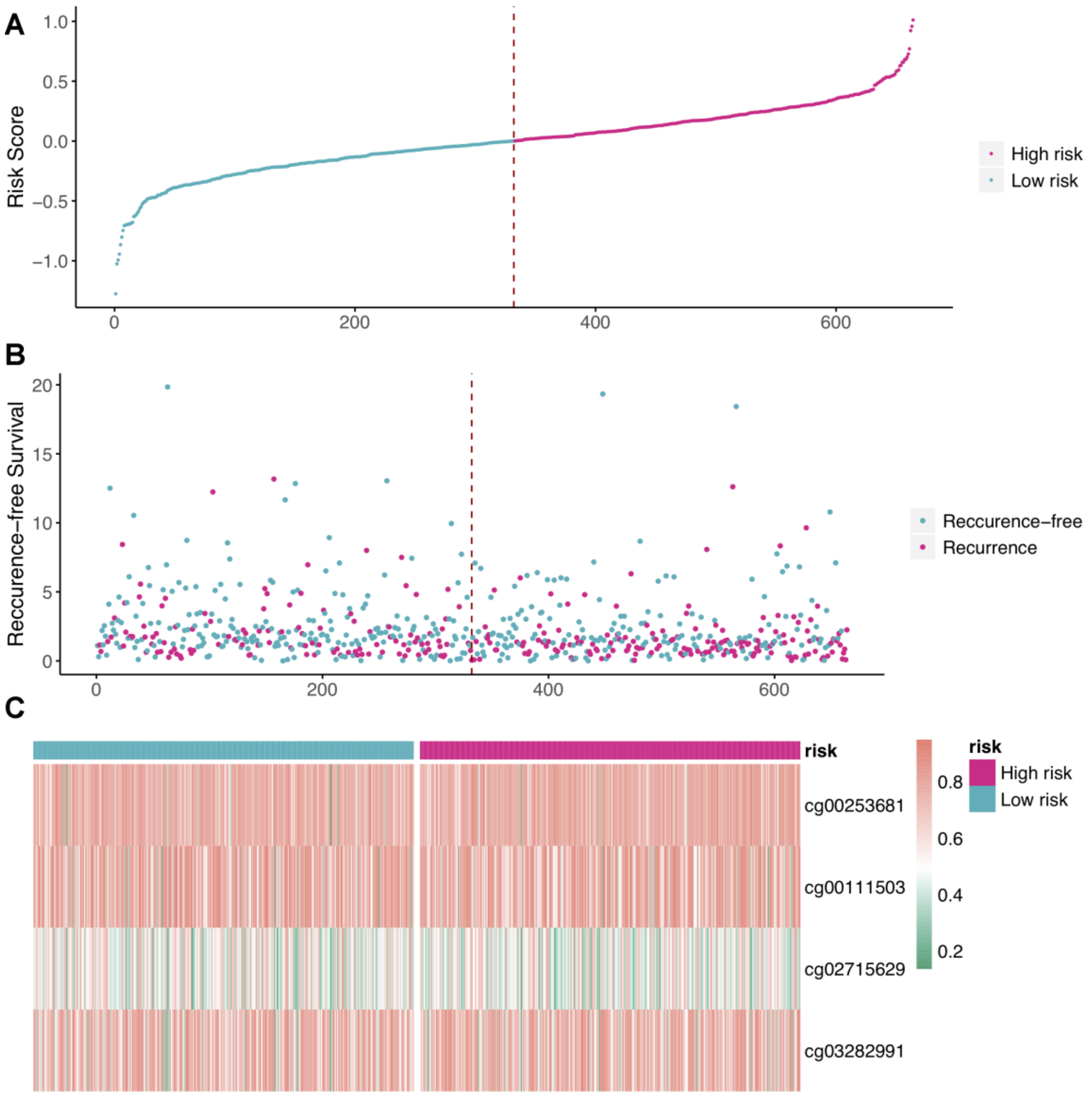

Supplementary Figure 2. Association chart of relapse risk factors. (**A**, **B**) Distribution of risk score and RFS of TCGA NSCLC patients in high- and low-risk groups. (**C**) The heatmap of DNAm profile of 4 final selected CpGs.

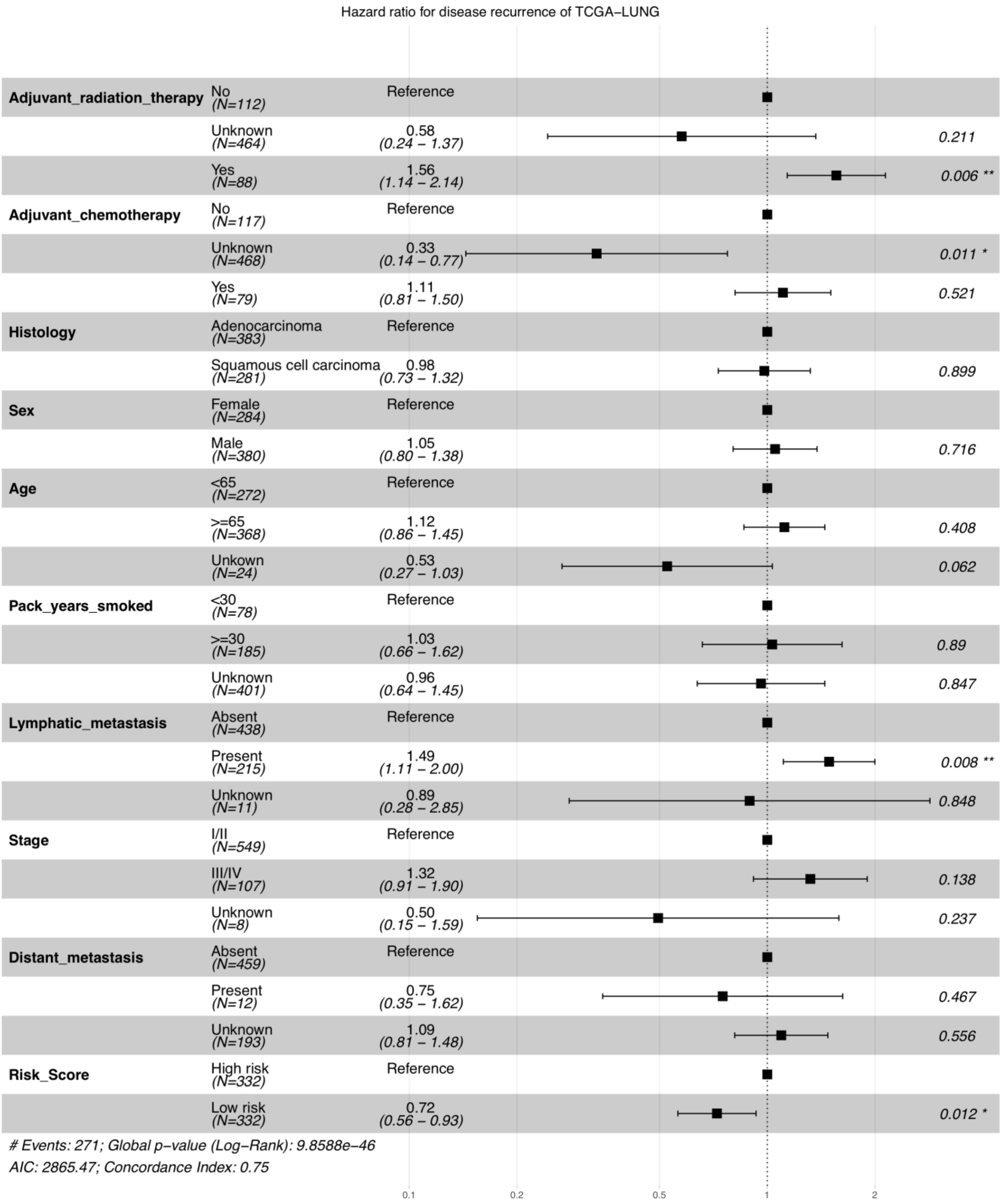

Supplementary Figure 3. Multivariate Cox regression analysis for RFS of TCGA NSCLC patients with combinations of DNAm-based risk score and clinical factors.

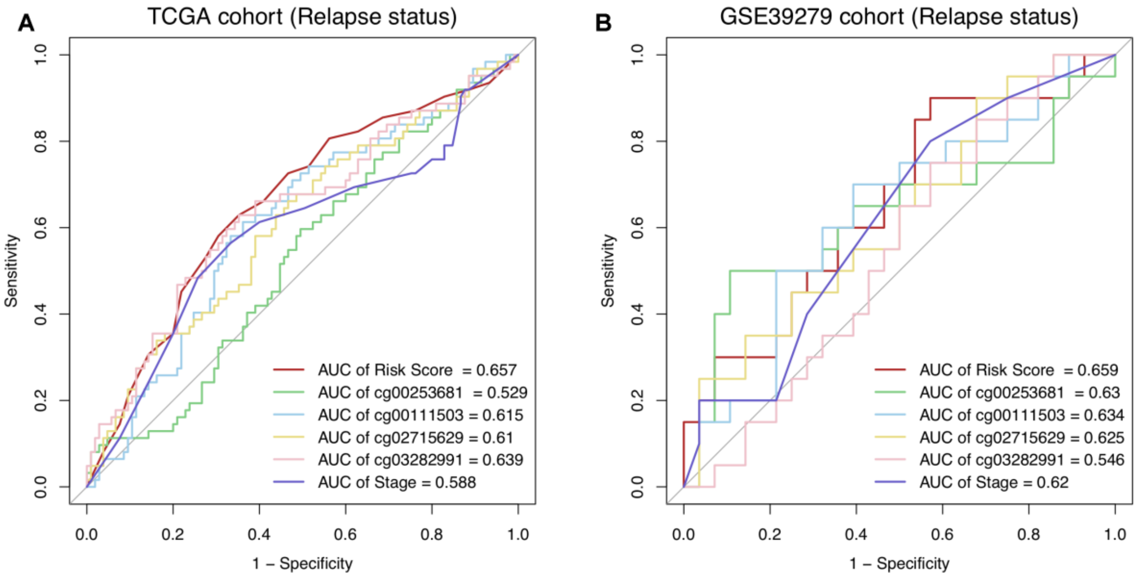

Supplementary Figure 4. Receiver operating characteristic (ROC) curves of the combined risk score, clinical stage and 4 separate CpGs demonstrate their performance of discrimination for relapse status in training (**A**) and validation (**B**) cohorts.

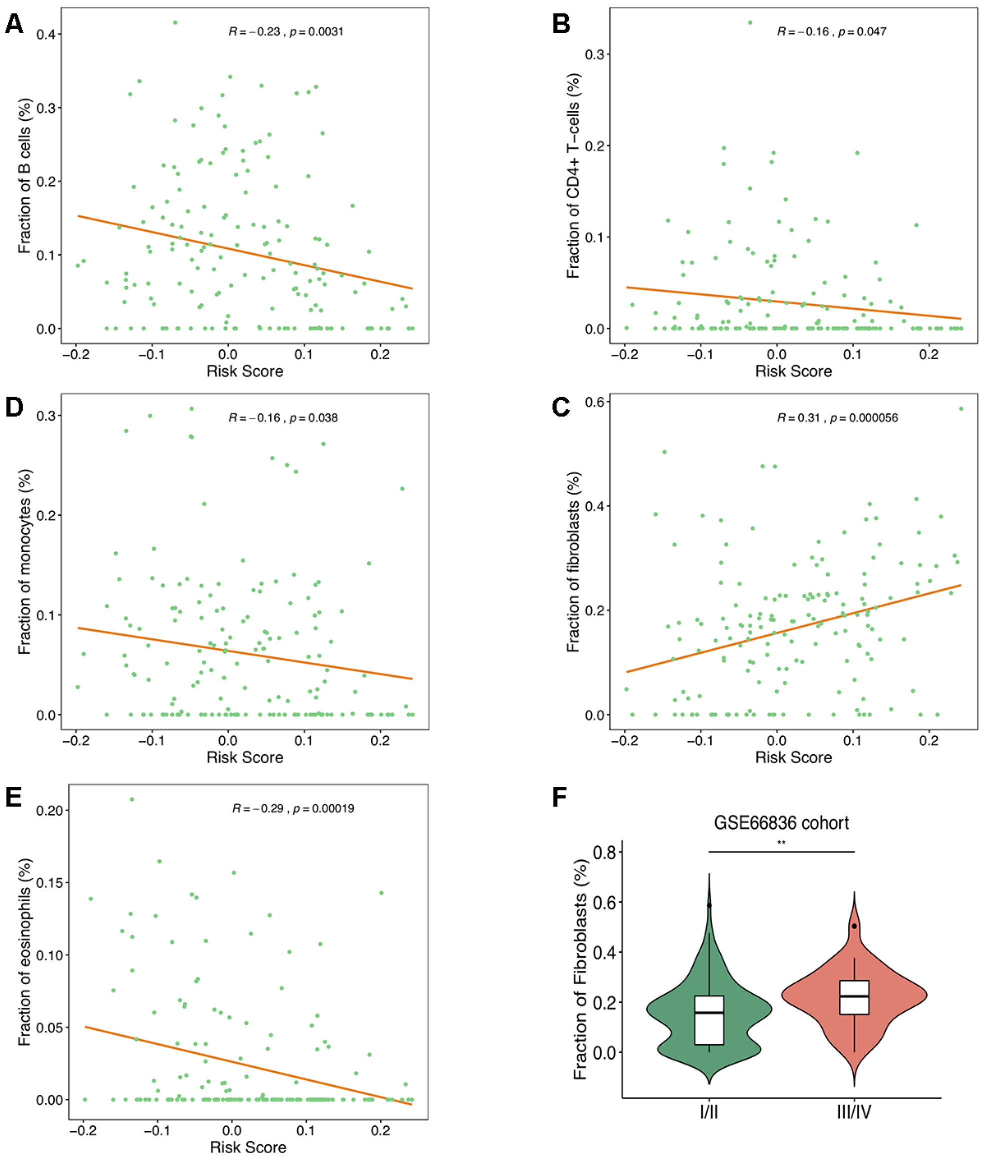

Supplementary Figure 5. Estimated compositions of B-cells (**A**), CD4+ T-cells (**B**), fibroblasts (**C**), monocytes (**D**) and eosinophils (**E**) were significantly correlated with DNAm-based risk score in GSE66836 cohort. (**F**) Fraction of fibroblasts in NSCLC samples at late stage was significantly higher than those at early stage.

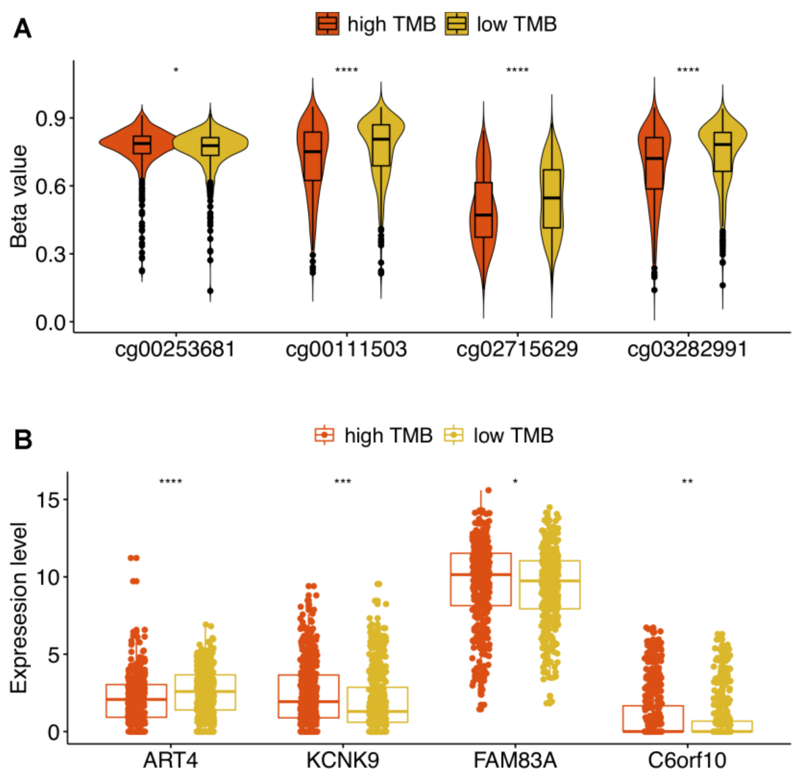

Supplementary Figure 6. The correlation of TMB with four CpGs methylation status and expression of four nearby genes. (**A**) Methylation levels of 4 identified DMPs in high TMB compared with low TMB group. (**B**) Differential expression of 4 reference genes in high and low TMB.
