# Supplementary material for: Identifying CpG methylation signature as a promising biomarker for recurrence and immunotherapy in non–small-cell lung carcinoma
Source: Aging (Albany NY). 2020 Jul 28;12(14):14649–76. doi: 10.18632/aging.103517 (PMC7425482; doi:10.18632/aging.103517)
Supplement: Supplementary Tables [file aging-12-103517-s001..pdf]

SUPPLEMENTARY TABLES

Supplementary Table 1. Clinical characteristics of patients for included study cohorts.

Characteristics		Training cohort (TCGA)	Validation cohort (GSE39279)	Validation cohort (GSE66836)	Validation cohort (GSE119144)
Total		n=823	n=444	n=164	n=60
Sex	Female	340	190	91	
	Male	483	254	73	
Age	<65	318	200		
	>=65	466	243		
	Unknown	39	1		
Pack-years smoked	<30	94	124		
	>=30	228	237		
	Unknown	501	83		
Histology	Adenocarcinoma	455	322	164	
	Squamous cell carcinoma	368	122	0	
Stage	I	420	237	93	
	II	244	94	40	
	III	127	102	29	
	IV	24	11	2	
	Unknown	8			
Lymphatic metastasis	Absent	534	239		
	Present	273	116		
	Unknown	16	89		
Distant metastasis	Absent	577	344		
	Present	23	11		
	Unknown	223	89		
Adjuvant radiation therapy	Yes	129	235		
	No	90	39		
	Unknown	604	170		
Adjuvant chemotherapy	Yes	136	250		
	No	79	24		
	Unknown	608	170		
Outcome(RFS)	Recurrence-free	399	150		10
	Recurrence	269	161		49
	Unknown	155	133		1
Outcome(OS)	Alive	502			
	Dead	321			
Follow up time(year/month)	Available(PFS)	662			59
	Unknown(PFS)	161			1
	Available(OS)	808			
	Unknown(OS)	15			

*RFS : recurrence-free survival status; OS : overall survival status.

Supplementary Table 2. Recurrence associated CpG markers identified by Univariate Cox, Random Forest and LASSO methods in training cohort.

Marker ID	Chr	Pos	Ref Gene	Location	logFC	adj.P	Method
cg00017489	chr7	153583318	DPP6	TSS1500	0.291	5.61E-39	LASSO-Logistic/Random Forest
cg00253681	chr12	14996583	ART4	TSS200	0.119	2.50E-21	LASSO-Logistic/Random Forest/Univariate Cox/LASSO-Cox
cg00682263	chr15	66188803	MEGF11	3'UTR	0.382	2.88E-87	LASSO-Logistic
cg02382109	chr8	22785456	PEBP4	TSS200	0.064	2.43E-06	LASSO-Logistic/Random Forest/LASSO-Cox
cg03389538	chr3	128779498	GP9	TSS200	0.043	7.37E-07	LASSO-Logistic/Random Forest
cg03502002	chr18	74962133	GALR1	1stExon;5'UTR	0.463	7.43E-52	LASSO-Logistic/Random Forest/LASSO-Cox
cg00111503	chr8	140631116	KCNK9	Body	-0.169	7.35E-20	LASSO-Logistic/Random Forest/Univariate Cox/LASSO-Cox
cg00814751	chr5	176072170	EIF4E1B	Body	-0.095	1.19E-10	LASSO-Logistic
cg01522296	chr22	50452415	IL17REL	TSS1500	-0.107	2.26E-13	LASSO-Logistic/Random Forest
cg02310286	chr8	88886432	DCAF4L2	TSS200	-0.141	9.58E-11	LASSO-Logistic/Random Forest/Univariate Cox
cg02407493	chr16	2068942	NPW	TSS1500	-0.148	5.53E-19	LASSO-Logistic/Random Forest/Univariate Cox
cg02715629	chr8	124193817	FAM83A	TSS1500;TSS1500	-0.247	1.75E-35	LASSO-Logistic/Random Forest/Univariate Cox/LASSO-Cox
cg02901006	chr19	8117024	CCL25	TSS1500	-0.117	1.57E-10	LASSO-Logistic/Random Forest
cg03282991	chr6	32294260	C6orf10	Body	-0.166	1.03E-17	LASSO-Logistic/Univariate Cox/LASSO-Cox
cg00446413	chr7	153749206	DPP6	TSS1500;Body	0.264	1.41E-50	Random Forest
cg02263813	chr16	56672640	MT1A	1stExon;5'UTR	0.233	2.38E-28	Random Forest
cg02099194	chr13	43149689	TNFSF11	Body;5'UTR	-0.153	6.42E-19	Random Forest
cg00914726	chr1	60539400	C1orf87	1stExon;5'UTR	0.233	3.82E-17	Random Forest
cg00472801	chr6	62995876	KHDRBS2	1stExon;5'UTR	0.132	8.77E-13	Random Forest
cg02096663	chr4	178650141	LOC285501	Body	-0.147	2.55E-10	Random Forest
cg00174500	chr14	23846479	CMTM5	1stExon;1stExon	0.045	0.000975951	Random Forest/LASSO-Cox
cg02062418	chr16	1494677	CCDC154	TSS200	-0.036	7.32E-06	Random Forest/LASSO-Cox
cg03322234	chr7	1022643	CYP2W1	TSS200	-0.033	0.046378826	Random Forest/LASSO-Cox
cg02992224	chr11	93822294	HEPHL1	Body	-0.123	1.25E-11	Random Forest
cg01466017	chr10	17496720	ST8SIA6	TSS1500	0.095	1.55E-07	Univariate Cox
cg03377767	chr2	17997138	MSGN1	TSS1500	-0.249	5.44E-47	Univariate Cox

*logFC: log2 fold change; adj.P: Benjamini-Hochberg adjusted P value.

Supplementary Table 3. Multivariable regression analysis for PFS of TCGA NSCLC patients conducted on clinical factors in combination with 13 biomarkers identified by LASSO-Cox and univariate Cox models.

**Characteristics**		**Coefficient**	**Hazard Ratio**
Adjuvant radiation therapy	Yes vs. No	0.461	1.59
	Unknown vs. No	-0.514	0.6
Adjuvant chemotherapy	Yes vs. No	0.206	1.23
	Unknown vs. No	-1.13	0.32
Histology	Squamous cell carcinoma vs. Adenocarcinoma	0.098	1.1
Sex	Male vs. Female	0.062	1.06
Age	>=65 vs. <65	0.17	1.19
	Unknown vs. <65	-0.552	0.58
Pack-years smoked	>=30 vs. <30	0.091	1.1
	Unknown vs. <30	-0.037	0.96
Lymphatic metastasis	Present vs. Absent	0.373	1.45
	Unknown vs. Absent	0.009	1.01
Stage	III/IV vs. I/II	0.347	1.41
	Unknown vs. I/II	-0.872	0.42
Distant metastasis	Present vs. Absent	-0.422	0.66
	Unknown vs. Absent	0.049	1.05
**cg00253681**	**Hypermethylation vs. Hypomethylation**	**0.339**	**1.4**
cg02382109	Hypermethylation vs. Hypomethylation	0.057	1.06
cg03502002	Hypermethylation vs. Hypomethylation	-0.27	0.76
**cg00111503**	**Hypermethylation vs. Hypomethylation**	**-0.337**	**0.71**
**cg02715629**	**Hypermethylation vs. Hypomethylation**	**-0.138**	**0.87**
**cg03282991**	**Hypermethylation vs. Hypomethylation**	**0.037**	**1.04**
cg00174500	Hypermethylation vs. Hypomethylation	-0.389	0.68
cg02062418	Hypermethylation vs. Hypomethylation	0.261	1.3
cg03322234	Hypermethylation vs. Hypomethylation	0.302	1.35
cg01466017	Hypermethylation vs. Hypomethylation	0.249	1.28
cg02310286	Hypermethylation vs. Hypomethylation	-0.18	0.84
cg02407493	Hypermethylation vs. Hypomethylation	0.064	1.07
cg03377767	Hypermethylation vs. Hypomethylation	-0.044	0.96

Supplementary Table 4. Multivariate Cox regression analysis for OS of TCGA NSCLC patients with combinations of DNAm-based risk score and clinical factors.

Characteristics		Hazard Ratio	CI	P Value
Adjuvant chemotherapy	Yes vs. No	0.71	0.48-1.06	0.094
	Unknown vs. No	0.43	0.2-0.93	0.032
Adjuvant radiation therapy	Yes vs. No	1.28	0.86-1.89	0.218
	Unknown vs. No	1.26	0.58-2.77	0.557
Age	>=65 vs. <65	1.23	0.96-1.57	0.095
	Unknown vs. <65	0.55	0.26-1.14	0.11
Distant metastasis	Present vs. Absent	1.72	0.96-3.09	0.069
	Unknown vs. Absent	1.17	0.88-1.56	0.278
Histology	Squamous cell carcinoma vs. Adenocarcinoma	1.17	0.9-1.52	0.232
Lymphatic metastasis	Present vs. Absent	1.53	1.17-2.02	0.002
	Unknown vs. Absent	1.58	0.69-3.61	0.279
Pack-years smoked	>=30 vs. <30	1.1	0.72-1.69	0.652
	Unknown vs. <30	1.08	0.73-1.6	0.707
Risk Model	High risk vs. Low risk	1.4	1.11-1.77	0.004
Sex	Male vs. Female	0.99	0.78-1.27	0.963
Stage	III/IV vs. I/II	1.41	1.02-1.96	0.04
	Unknown vs. I/II	0.95	0.3-3.03	0.935

*CI: 95% confidence interval.
